# SERPIND1 Affects the Malignant Biological Behavior of Epithelial Ovarian Cancer via the PI3K/AKT Pathway: A Mechanistic Study

**DOI:** 10.3389/fonc.2019.00954

**Published:** 2019-10-04

**Authors:** Qian Guo, Liancheng Zhu, Caixia Wang, Shuang Wang, Xin Nie, Juanjuan Liu, Qing Liu, Yingying Hao, Xiao Li, Bei Lin

**Affiliations:** ^1^Department of Obstetrics and Gynecology, Shengjing Hospital of China Medical University, Shenyang, China; ^2^Key Laboratory of Maternal-Fetal Medicine of Liaoning Province, Benxi, China; ^3^Key Laboratory of Obstetrics and Gynecology of Higher Education of Liaoning Province, Benxi, China

**Keywords:** epithelial ovarian cancer, SERPIND1, prognosis, proliferation, cell cycle, cell migration, EMT, NF-κB1

## Abstract

Serpin family D member 1 (SERPIND1) belongs to the serine protease inhibitor family. Its role in cancers has gradually attracted interest from researchers in recent years. However, the role of SERPIND1 in the development of epithelial ovarian cancer remains poorly understood. This studied aimed to investigate the expression and clinical significance of SERPIND1 in epithelial ovarian cancer, as well as its effect on the malignant biological behavior of ovarian cancer cells and the related regulatory mechanisms. We found that SERPIND1 expression was significantly elevated in epithelial ovarian cancer. Patients with higher expression of SERPIND1 in ovarian cancer tissues had poor prognoses. SERPIND1 promoted the proliferation, migration, invasion, G1-to-S phase transition, and epithelial–mesenchymal transition of ovarian cancer cells and inhibited their apoptosis by promoting phosphorylation in the phosphoinositide 3-kinase/protein kinase B (PI3K/AKT) pathway. Meanwhile, the inhibition of SERPIND1 expression in ovarian cancer cells resulted in opposite effects. The addition of the PI3K/AKT pathway inhibitor LY294002 to SERPIND1-overexpressing cells could reverse the promoting effect of SERPIND1 on the malignant biological behavior of ovarian cancer cells. Further, nuclear factor kappa B subunit 1, a transcription factor could bind to the promoter region of SERPIND1 and regulate SERPIND1 expression. In conclusion, our results indicated that SERPIND1 could be an effective marker for assessing the prognosis of ovarian cancer. By elucidating its mechanism underlying the promotion of malignant biological behavior of ovarian cancer by SERPIND1, we demonstrated that SERPIND1 could potentially serve as a novel drug target.

## Introduction

Ovarian cancer is a common malignancy in females and has one of the highest mortality rates. It accounts for 2.5% of all female malignancies and 5% of female cancer mortality. These rates are primarily caused by late-stage diagnoses (1). Early-stage ovarian cancer generally lacks obvious symptoms (2) and specific cancer biomarkers (3, 4). Between 61 and 75% of ovarian cancers are not discovered until they have reached an advanced stage (stage III or later) (1, 5). Due to the poor prognosis of ovarian cancer, the 5-year survival rate of patients with advanced disease is <47% (5, 6). Epithelial ovarian cancers are the most common ovarian cancers, accounting for 90% of all ovarian malignancies (1). Therefore, the search for specific cancer biomarkers has become popular in ovarian cancer research, as it is of great significance for the early diagnosis and prognostication of the disease and for the development of new therapeutic targets.

Serine proteinase inhibitors (Serpins) are a family of highly conserved protease inhibitors that are widely distributed in mammals, birds, plants, bacteria, and viruses (7). Early studies have shown that serpins possess inhibitory activity against serine proteases. Serpins also appear to play a role in immune function, blood clotting, dementia, tumorigenesis, and metastasis. For instance, serpin family A member 7 (SERPINA7) and serpin family H member 1 (SERPINH1) do not inhibit proteases, but they participate in the transport of hormones and the folding of proteins (8–13). SERPINB3 displays an antiapoptotic behavior, and alterations in its expression might contribute to apoptotic dysregulation (9). Several members of the serpin family have also been found to play a critical role in cancer development. For example, SERPINA9 is abundantly expressed in B-cell lymphomas (14), and SERPINB5 expression is elevated in epithelial ovarian cancers (15). Additionally, SERPINB5 can promote the metastasis of pancreatic cancer and breast cancer (16, 17). SERPINI2 may also promote the metastasis of pancreatic cancers (18).

SERPIND1 can inhibit thrombin activity through interaction with heparin; hence, it is also known as heparin cofactor II (19). SERPIND1 can also promote the release of leukocyte chemotactic factors and induce angiogenesis (20, 21). Its role in cancers has gradually attracted interest from researchers in recent years. Liao et al. found that SERPIND1 was overexpressed in non-small cell lung cancer (NSCLC), and that patients whose cancer tissues had higher SERPIND1 expression experienced a higher recurrence rate and a shorter overall survival than patients with lower SERPIND1 expression (22). They also overexpressed and inhibited SERPIND1 expression in human lung cancer cell lines and found that SERPIND1 could promote the formation of pseudopodia in NSCLC cells and increase cell motility. Another study found that SERPIND1 expression is upregulated in acute lymphoblastic leukemia, and SERPIND1 may be a candidate cancer biomarker for diagnosing the disease (23).

The research on SERPIND1 in cancers is limited to NSCLC and leukemia (22, 23), and studies of SERPIND1 in other cancer types have not yet been conducted. This present study aimed to examine the expression of SERPIND1 in epithelial ovarian cancers and further analyze its relationship with clinicopathological parameters. The study also investigated the effect of SERPIND1 on the malignant biological behavior of ovarian cancer cells and the related regulatory mechanisms.

## Materials and Methods

### Patients and Source of Pathological Tissues

Between 2008 and 2012, surgically resected, paraffin-embedded specimens from 197 patients at the Department of Obstetrics and Gynecology of Shengjing Hospital were obtained. Among 125 patients with malignant ovarian cancer, 113 were followed up through the collection of clinical information. After surgery, patients were followed up once every 6 months.

Diagnoses were reevaluated and confirmed in all tissue sections by pathologists. According to the results, there were 77, 18, 8, and 10 cases of serous carcinoma, mucinous carcinoma, endometrioid carcinoma, and clear-cell carcinoma, respectively. The median age of the patients was 53 years.

The following median ages were reported: for 113 patients with epithelial ovarian cancers, 53 years; for 24 patients with borderline ovarian tumors, 41 years; for 24 patients with benign ovarian tumors, 45.5 years; and for 24 patients in the normal group (normal ovarian specimens resected during surgery for cervical cancer), 45 years. There was no statistically significant difference in age among the groups (*P* > 0.05). Regarding pathological grade, the malignant group had 68, 33, and 12 patients with poorly differentiated, moderately differentiated, and well-differentiated ovarian cancers, respectively. In terms of surgical–pathological staging according to the International Federation of Gynecology and Obstetrics (FIGO) criteria, 6, 63, 12, and 32 patients had stage IV, III, II, and I disease, respectively. Among the 113 patients in the malignant group, 17 had lymph node metastasis; 73 of these 113 patients underwent lymph node dissection during operation, while 40 had no lymph node dissection during surgery. All patients were newly diagnosed, and their clinical data were complete, including data on age, surgical staging, lymph node metastasis, pathological type, and degree of differentiation. All patients had not received chemotherapy or radiotherapy before surgery.

### Immunohistochemistry

The ovarian tissues from each specimen were prepared as 4 μm serial sections. SERPIND1 expression was detected with immunohistochemistry using a streptavidin–peroxidase conjugate. Positive and blank controls were prepared for each batch of sections. Positive controls were prepared from tissues (liver cancer tissues) that had been confirmed positive from previous experiments, and blank controls were prepared with phosphate buffer in place of the primary antibody. The working concentration of the rabbit anti-human SERPIND1 antibody was 1:400.

### Criteria for Immunohistochemical Staining

Positive staining was defined as the presence of buff-colored granules in the cytoplasm and nucleus. Immunohistochemical assays and staining was scored as previously reported (24). A score of ≤2 indicated negative expression, while >2 indicated positive expression. At the same time, a score of ≤4 indicated weak expression, while >4 indicated strong expression. The results were obtained by two blinded observers who independently observed each section and performed the cell counting and background evaluation. In case of disagreement on the results, a third observer would make the final decision.

### Western Blotting

The experimental method was performed as previously described (24). The primary antibodies used in our study were as follows: SERPIND1 (1:1000 dilution; Abcam, Cambridge, UK); E-cadherin, N-cadherin, MMP2, and MMP9 (1:1000 dilution; Proteintech Group Inc., USA); Vimentin (1:1500 dilution; Proteintech Group Inc., USA); and PI3K p85, phospho-PI3K p85(Tyr458)/p55(Tyr199), AKT (pan), and phosphor-AKT (Ser473) (1:1000 dilution; Cell Signaling Technology, Danvers, MA, USA); GAPDH (1:3000 dilution; Zhong Shan Company, China). The proteins were developed using an enhanced chemiluminescence reagent (Millipore, USA). See Supplementary Material for more details.

### Cell Culture and Transfection

Human ovarian cancer cell lines CAOV3, OVCAR3, and SKOV3 were conventionally cultured in Roswell Park Memorial Institute (RPMI) 1640 medium containing 10% fetal bovine serum, while ES-2 cells were cultured in McCoy's 5A medium containing 10% fetal bovine serum. All cells were cultured at 37°C in a saturated-humidity environment containing 5% CO_2_. SERPIND1 expressed in all human ovarian cancer cell lines, with lower expression in ES-2 and higher expression in CAOV3 and OVCAR3. Therefore, we decided to inhibit the expression of the *SERPIND1* gene using siRNA technology in CAOV3 and OVCAR3 cells. We also overexpressed the *SERPIND1* gene using lentiviral transfection in ES-2 cells, and a stable ES-2 cell line that overexpressed SERPIND1 was established. The CAOV3 and OVCAR3 cells were transfected with SERPIND1-specific small-interfering RNA (siRNA) using Lipofectamine 3000 (Thermo Fisher Scientific, USA) according to the instruction manual. The sequence of the SERPIND1-Specific siRNA was GCACCCGUUG-GCAUUUCUATT, and the control sequence was UUCUCCGAACGUGUCACGUTT. The siRNAs were purchased from Gene Pharma (Shanghai, China). Lentivirus overexpression plasmids harboring SERPIND1 (LV5-SERPIND1) were purchased from Gene Pharma (Shanghai, China) and were transfection at a MOI of 10. The ES-2 cells were transfected with SERPIND1-coated lentivirus and empty controls using polybrene according to the instruction manual. The cells were then cultured with 2 μg/ml of puromycin for 1 week. RT-PCR was used to verify the cell lines with stable SERPIND1 overexpression. To inhibit PI3K/AKT signaling, the cells were treated with a PI3K inhibitor, LY294002 (10 μM; Selleck, Houston, USA) for 48 h.

### *In vivo* Tumorigenesis Model

All nude mice were purchased from HFK Bioscience Co, Ltd (Beijing, China). A total of 2 × 10^6^ SERPIND1-overexpressing ES-2 cells and ES-2-control cells were injected subcutaneously or intraperitoneally into 5-week-old BALB/c female nude mice. The body weight of the nude mice and tumor volume were measured every 5 days. The width (W) and length (L) of the tumor were measured by a caliper (tumor volume, cm^3^ = 0.5 × L × W^2^) (25). At day 26 after tumor cell implantation, the mice were euthanized. The tumors were excised, measured, and imaged.

### Scratch-Wound Assay

The transfected cells were cultured for 48 h and then seeded into 6-well plates at a density of 1 × 10^6^ cells/ml. When the cell reached 90% confluence, vertical straight lines were drawn in the wells (with the same diameter throughout all wells) using a 200 μl pipette tip, with the long axis of the tip being perpendicular to the bottom of the well. The detached cells were removed with phosphate-buffered saline, and images were captured at 0 and 24 h under a microscope. Wound healing rate was calculated as follows: (original wound area—wound area after 24 h)/original wound area × 100%.

### Invasion Assay

For the migration capacity test, 70 μl of Matrigel (BD Biosciences, New Jersey, USA) was placed in the upper chamber of the Transwell (Corning Costar Corporation, Cambridge, MA) and air-dried. The transfected cells were cultured for 48 h and then trypsinized and seeded into 6-well plates at a density of 1 × 106 cells/ml. Then, 200 μl of cells was added into the upper chamber of the Transwell, and 600 μl of culture medium containing 20% fetal bovine serum was added into the lower chamber. The cells were cultured for 24 h. Invasion assays and staining were scored as previously reported (26). After being rinsed with phosphate-buffered saline, the cells were observed under a microscope, and five fields of view were randomly selected for counting the cells.

### Cell Proliferation Assay

MTT assays were used to determine the association between SERPIND1 expression and changes in cell proliferative capacity. CAOV3 and OVCAR3 cells were transfected with SERPIND1 siRNA and blank control; ES-2 cells were transfected with SERPIND1-expressing vectors and empty control. The cells were seeded into 96-well plates and incubated. Cell proliferation was evaluated every 24 h. In each well, 20 μl of MTT (Solarbio, Beijing, China) solution was added to the culture medium and incubated in the dark for 4 h, after which the solution was carefully aspirated from the culture medium. Dimethyl sulfoxide was added to each well, and the mixture was shaken at a low speed for 9 min on a shaker to fully dissolve the crystals. The absorbance value was measured at 490 nm using a microplate reader.

### Apoptosis Assay

The cells were harvested 48 h after transfection, resuspended at a density of 1 × 10^6^ cells/ml, and mixed with 500 μl of 1 × buffer and 5 μl of Annexin-V- FITC/PI or Annexin V-PE/7-AAD (BD Biosciences, New Jersey, USA). The cells were incubated in the dark for 60 min, and the apoptotic rate was measured using flow cytometry.

### Cell Cycle Analysis

The cells were digested with EDTA-free trypsin, harvested, and washed twice with phosphate-buffered solution. Other experimental steps were performed as previously described (26). Propidium iodide signals were examined using flow cytometry. Approximately 2 × 10^6^ cells were evaluated for each sample.

### Chromatin Immunoprecipitation

Chromatin immunoprecipitation assays were performed using a commercially available Chromatin immunoprecipitation kit (9004; Cell Signaling Technology, Danvers, MA, USA). The experimental steps were as previously described (27). Briefly, sonicated chromatin solutions were incubated at 4°C overnight with anti-nuclear factor kappa B1 (NF-κB1) antibody (1:50 dilution; Cell Signaling Technology, Danvers, MA, USA), anti-RNA polymerase II (positive control), and normal mouse IgG (negative control). Then, DNA and the transcription factor complex were crosslinked, and a purified DNA fragment was obtained. PCR was performed to verify the binding of NF-κB1 at the promoter region of SERPIND1. PCR was performed using the primers spanning the putative NF-κB1 binding sites in the promoter region of human SERPIND1 (forward 5′-CAAGATTTAACCAGCCAGTC-3′, reverse 5′-AACCCACAGAAAGCATCA-3′; forward 5′-GGCGTTACTCTTCTTGAC-3′; reverse 5′-CTCCCACCCACTAAACTG-3′).

### Statistical Analysis Ethics Approval and Informed Consent

Student's *t*-test was used to assay the significant differences between the experimental and control groups. Pearson's χ^2^-test was used for clinically relevant experiments. The mean values are representative of at least 3 independent experiments. All the results are expressed as the mean ± standard deviation (SD). Statistical analysis was performed using SPSS 21.0 and GraphPad Prism 7.0. *P* ≤ 0.05 was considered statistically significant.

### Ethics Approval and Informed Consent

The use of nude mice in this study was approved by the Medical Ethics Committee of Shengjing Hospital of China Medical University (2018PS106K). Pathological analysis was approved by the Ethics Review Committee of China Medical University (2010PS84K).

## Results

### Expression of SERPIND1 in Different Ovarian Tissues

**Figure 1:**
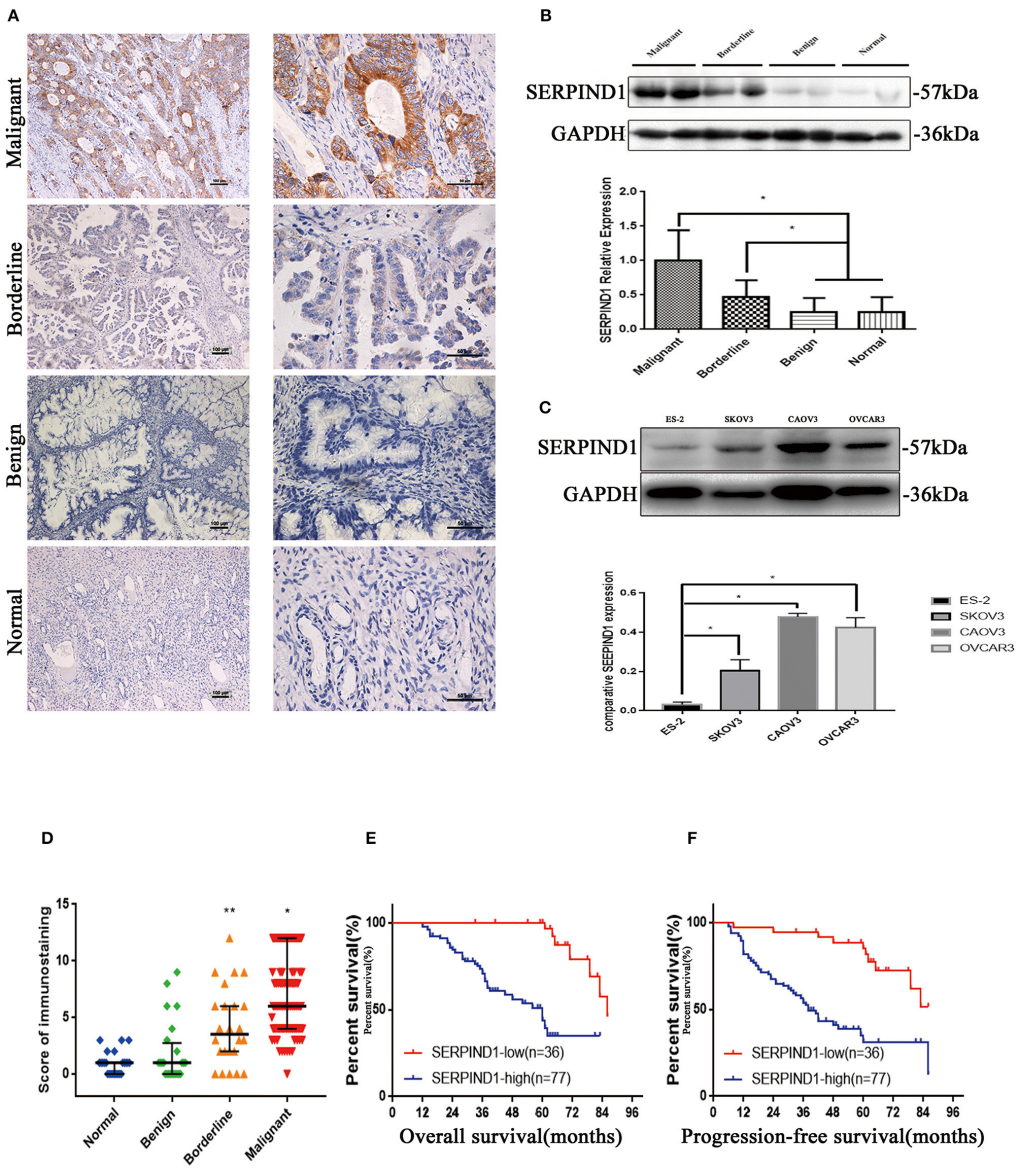
SERPIND1 expression was upregulated in ovarian cancer. **(A)** Results of immunohistochemistry: expression of the SERPIND1 protein in tissues from malignant, borderline, and benign epithelial ovarian tumors and normal ovarian tissues. Left scale bar: 100 μm. Right scale bar: 50 μm. **(B)** Results of Western blot: expression of the SERPIND1 protein in tissues from malignant, borderline, and benign epithelial ovarian tumors and normal ovarian tissues. **(C)** Expression of SERPIND1 in ovarian cancer cell lines. **(D)** SERPIND1 expression levels assessed by immunohistochemistry and plotted as medians with interquartile ranges. **(E)** On Kaplan–Meier analysis, overall survival was significantly higher in the low SERPIND1 expression group than in the high SERPIND1 expression group. **(F)** On Kaplan–Meier analysis, progression-free survival was substantially higher in the low SERPIND1 expression group than in the high SERPIND1 expression group. Data are presented as mean ± standard deviation from three independent experiments. *P < 0.05, **P < 0.05. SERPIND1, serpin family D member 1.

Immunohistochemistry results showed that SERPIND1 was primarily located in the cytoplasm, with a small amount in the nucleus. The majority of the staining showed evenly distributed yellow or yellow-brown granules or sheets. Positive and strongly positive rates of SERPIND1 expression were reported for the malignant group (90.40 and 67.20%, respectively), the malignant group included 77 serous carcinomas, 18 mucinous carcinomas, 8 endometrioid carcinomas, and 10 clear-cell carcinomas. Positive and strongly positive rates of SERPIND1 expression were reported for the borderline group (62.50 and 37.50%, respectively), benign group (20.83 and 16.67%, respectively), and normal ovarian tissue group (12.50 and 0.00%, respectively). SERPIND1 was highly expressed in the malignant group, compared with the borderline, benign, and normal groups (*P* < 0.05 in all cases). Moreover, SERPIND1 expression was higher in the borderline group than in the benign and normal groups (*P* < 0.05 in all cases). Furthermore, SERPIND1 expression was higher in the benign group than in the normal group, but the difference was not statistically significant (*P* > 0.05) (Figure 1A).

Western blot was used to examine SERPIND1 expression in ovarian tissues in 27, 24, 8, and 9 cases of malignant, borderline, benign, and normal ovarian tissues (normal ovarian specimens resected during surgery for cervical cancer), respectively. As shown in Figure 1B, SERPIND1 expression in tissues from the malignant and borderline groups was higher than that from the benign and normal groups (*P* < 0.05 in all cases). The expression rate of SERPIND1 was higher in the benign group than in the normal group, but the difference was not statistically significant (*P* > 0.05).

### Expression of SERPIND1 in Ovarian Cancer Cells

We also examined SERPIND1 expression in four human ovarian cancer cell lines: CAOV3 (human ovarian adenocarcinoma cells), OVCAR3 (human ovarian adenocarcinoma cells), ES-2 (human ovarian clear-cell carcinoma cells), and SKOV3 (human serous ovarian cancer cells). The results showed that SERPIND1 was expressed in all four human ovarian cancer cell lines, with lower expression in ES-2 and higher expression in CAOV3 and OVCAR3 (Figure 1C). Therefore, we decided to inhibit the expression of the *SERPIND1* gene using siRNA technology in CAOV3 and OVCAR 3 cells. We also overexpressed the SERPIND1 gene using lentiviral transfection in ES-2 cells, and a stable ES-2 cell line that overexpressed SERPIND1 was established.

### Relationship Between SERPIND1 and Clinicopathological Parameters in Epithelial Ovarian Cancers

We analyzed the data of the 113 patients with primary epithelial ovarian cancers and compared the clinicopathological parameters with SERPIND1 tissue expression (Table 1). The analyses showed SERPIND1 expression in tissues from the malignant and borderline groups was higher than that from the benign and normal groups (Figure 1D, *P* < 0.05). And the positive and strongly positive expression rates of SERPIND1 were higher in ovarian serous cystadenocarcinoma than in ovarian mucinous cystadenocarcinoma (97.4 and 72.7% vs. 77.8 and 44.4%, *P* < 0.05). However, differences among other types of epithelial ovarian cancers were not significant. The strongly positive expression rate of SERPIND1 was significantly higher in FIGO stage III–IV epithelial ovarian cancers than in stage I–II epithelial ovarian cancers (85.1% vs. 43.5%, *P* < 0.01). Positive and strongly positive expression rates of SERPIND1 were reported for the poorly differentiated group (97.1 and 67.6%, respectively), the moderately differentiated group (90.9 and 75.8%, respectively), and the well-differentiated group (83.3 and 50%, respectively). SERPIND1 expression increased with decrease in the degree of differentiation, but the trend was not significantly different. Meanwhile, positive and strongly positive expression rates of SERPIND1 were higher in the lymph node metastasis–positive group than in the lymph node metastasis–negative group, but the differences were not statistically significant (100 and 82.4% vs. 94.6 and 76.8%, *P* > 0.05 in all cases).

**Table 1:** Relationships between the positive rate of SERPIND1 expression and clinicopathological characteristics of patients with malignant epithelial ovarian tumors.

**Characteristics**	**No**.	**SERPIND1 expression**					
	**Cases**	**Negative (–)**	**Positive** **(+ ~ +++)**	***P-*value**	**Weakly positive** **(±)**	**Strongly positive (++/+++)**	***P*-value**
Pathologic type				*P* < 0.05			*P* < 0.05
Serous	77	2 (2.6%)	75 (97.4%)^*^		21 (27.3%)	56 (72.7%)^**^	
Mucinous	18	4 (22.2%)	14 (77.8%)		10 (55.6%)	8 (44.4%)	
Endometrioid	8	0 (0.0%)	8 (100.0%)		1 (12.5%)	7 (87.5%)	
Clear cell carcinoma	10	1 (10%)	9 (90%)		4 (40%)	6 (60%)	
Surgical stage				*P* = 0.119			*P* < 0.01
I–II	46	5 (10.9%)	41 (89.1%)		26 (56.5%)	20 (43.5%)	
III–IV	67	2 (3.0%)	65 (97.0%)		10 (14.9%)	57 (85.1%)	
Differentiation				*P* = 0.137			*P* = 0.258
Well	12	2 (16.7%)	10 (83.3%)		6 (50%)	6 (50%)	
Moderate	33	3 (9.1%)	30 (90.9%)		8 (24.2%)	25 (75.8%)	
Poor	68	2 (2.9%)	66 (97.1%)		22 (32.4%)	46 (67.6%)	
Lymph node metastasis^a^				*P* = 0.330			*P* = 0.627
No	56	3 (5.4%)	53 (94.6%)		13 (23.2%)	43 (76.8%)	
Yes	17	0 (0.0%)	17 (100.0%)		3 (17.6%)	14 (82.4%)	

a *Forty patients did not undergo lymph node dissection*.

* *P < 0.05*,

** *P < 0.05*.

### Analysis of Prognostic Risk Factors in Epithelial Ovarian Cancers

Cox regression was used to analyze the effects of FIGO stage, SERPIND1 expression, pathological type, lymph node metastasis, degree of differentiation, and other factors on postoperative survival and recurrence. We found that FIGO stage and SERPIND1 expression were independent risk factors of prognosis of epithelial ovarian cancers (Table 2).

**Table 2:** The Univariate and multivariate Cox proportional analysis of disease-free survival of cases with ovarian carcinoma.

**Variables**	**Univariate analysis**		***P*-value**	**Multivariate analysis**		***P*-value**
	**HR**	**95% CI**		**HR**	**95% CI**	
FIGO stage (I + II vs. III + IV)	4.243	1.888 ~ 9.533	<0.001	2.487	1.048 ~ 5.902	0.039
SERPIND 1 (low *vs*. high)	5.584	2.416 ~ 12.906	<0.001	3.809	1.565 ~ 9.267	0.003
Differentiation (well-moderate vs. poor)	1.256	0.678 ~ 2.325	0.468	1.113	0.596 ~ 2.079	0.736
Lymph node metastasis (no vs. yes)	1.366	0.649 ~ 2.878	0.412	0.913	0.429 ~ 1.943	0.812
Age (≥53 vs. <53)	1.276	0.696 ~ 2.339	0.430	1.322	0.718 ~ 2.433	0.370

*HR, Hazard ratio; CI, confidence interval*.

### Survival Analysis

Results of the long-term follow-up showed that 7 deaths occurred in the low SERPIND1 expression group (*n* = 36), compared with 37 deaths in the high SERPIND1 expression group (*n* = 77). In the Kaplan–Meier analysis, the overall survival of the low SERPIND1 expression group was significantly higher than that of the high SERPIND1 expression group (*P* < 0.001, Figure 1E).

We also analyzed the relationship between SERPIND1 expression and progression-free survival in epithelial ovarian cancer. There were 48 patients who showed relapse in the high SERPIND1 expression group, whereas only 10 patients showed relapse in the low SERPIND1 expression group. The recurrence rate was substantially higher in the high SERPIND1 expression group than in the low SERPIND1 expression group (*P* < 0.001, Figure 1F).

### SERPIND1 Promoted Migration and Invasion Capacity of Ovarian Cancer Cells *in vitro*

**Figure 2:**
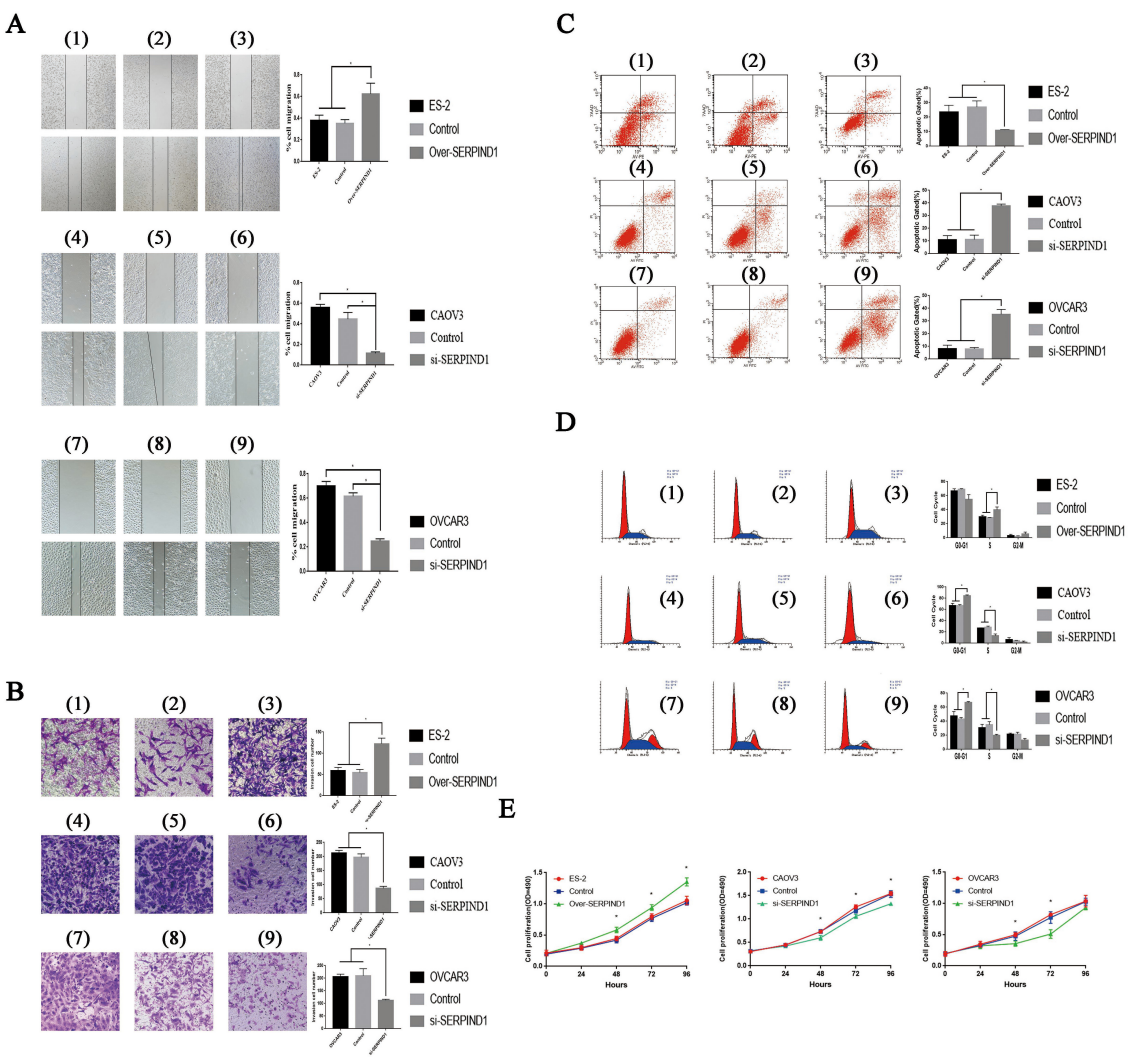
Effects of SERPIND1 on ovarian cancer cells *in vitro*. **(A)** SERPIND1 promoted the migration capacity of ovarian cancer cells. **(B)** SERPIND1 promoted the invasion capacity of ovarian cancer cells. **(C)** SERPIND1 inhibited apoptosis in ovarian cancer cell lines. **(D)** Effects of SERPIND1 on the cell cycle of ovarian cancer cell lines. **(E)** SERPIND1 promoted the proliferation of ovarian cancer cells. (1): ES-2; (2): ES-2-SERPIND1-H-Mock; (3): ES-2-SERPIND1-H; (4): CAOV3; (5): CAOV3-SERPIND1-L- Mock; (6): CAOV3-SERPIND1-L; (7): OVCAR3; (8): OVCAR3-SERPIND1-L- Mock (9): OVCAR3-SERPIND1-L. Data are presented as mean ± standard deviation from three independent experiments. *P < 0.05. Scale bar: 100 μm. SERPIND1, serpin family D member 1.

The scratch-wound assay and Transwell experiments were used to study the effects of SERPIND1 on the migration and invasion of ovarian cancer cells. Compared with the control cells, the migration and invasion capacities of SERPIND1-overexpressing ES-2 cells were significantly higher. In CAOV3 and OVCAR3 cells in which SERPIND1 expression was inhibited, the migration and invasion capacities were significantly lower than those of the control cells (Figures 2A,B). These results indicate that SERPIND1 promoted the migration and invasion of ovarian cancer cells.

### SERPIND1 Inhibited Apoptosis in Ovarian Cancer Cell Lines *in vitro*

Flow cytometry was used to detect changes in apoptosis before and after the differential expression of SERPIND1. Compared with the control cells, the apoptotic rate of SERPIND1-overexpressing ES-2 cells were significantly lower. In CAOV3 and OVCAR3 cells in which SERPIND1 expression was inhibited, the apoptotic rate was significantly higher than that of the controls (Figure 2C). These findings indicate that SERPIND1 inhibited the apoptosis of human ovarian cancer cells.

### Effects of SERPIND1 on the Cell Cycle of Ovarian Cancer Cell Lines *in vitro*

We examined cell-cycle changes before and after the differential expression of SERPIND1 in ovarian cancer cells. The results showed that after the overexpression of the *SERPIND1* gene, the proportion of S-phase cells was higher in ovarian cancer cells than in the control groups. After the inhibition of *SERPIND1* gene expression in CAOV3 cells, the proportion of S-phase cells was significantly lower in the CAOV3 cells than in the control and no-treatment groups. After the inhibition of *SERPIND1* gene expression in OVCAR3 cells, the proportion of S-phase cells was also significantly lower in the OVCAR3 cells than in the controls (Figure 2D). These results indicate that SERPIND1 promoted S- phase entry in ovarian cancer cells.

### SERPIND1 Promoted the Proliferation of Ovarian Cancer Cell Lines *in vitro*

The effect of SERPIND1 on the proliferation of ovarian cancer cells was studied using the MTT assay. The proliferation rate of SERPIND1-overexpressing ES-2 cells was accelerated compared with that of the control group. In CAOV3 and OVCAR3 cells in which SERPIND1 expression was inhibited, the proliferative capacity was significantly lower than in the controls (Figure 2E). These results indicate that SERPIND1 promoted the proliferative capacity of ovarian cancer cells.

### SERPIND1 Promoted the Proliferation and Peritoneal Metastasis of Ovarian Cancer Cell Lines *in vivo*

**Figure 3:**
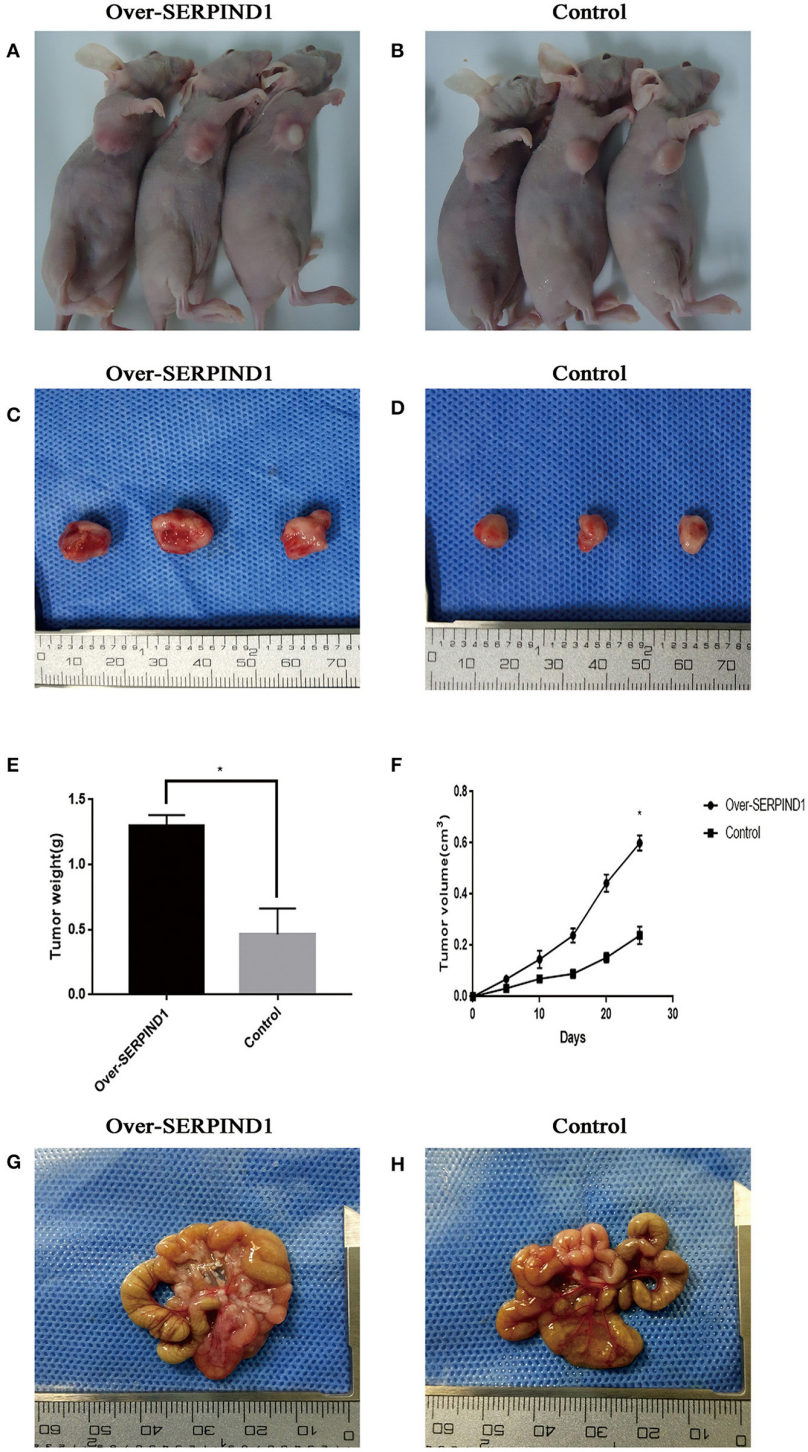
SERPIND1 promoted the proliferation and peritoneal metastasis of ovarian cancer cells *in vivo*. **(A)** Subcutaneous tumors developed from SERPIND1-overexpressing cells in nude mice. **(B)** Subcutaneous tumors developed from control cells in nude mice. **(C)** Tumors developed from SERPIND1-overexpressing cells. **(D)** Tumors developed from control cells. **(E)** Tumor weight in both groups. **(F)** Tumor volume in both groups. **(G)** Representative images of peritoneal metastasis; tumors developed from SERPIND1-overexpressing cells. **(H)** Representative images of peritoneal metastasis; tumors developed from control cells. Data are presented as mean ± standard deviation from three independent experiments. *P < 0.05. SERPIND1, serpin family D member 1.

The stable SERPIND1-overexpressing cells (Figures 3A,C) and the corresponding empty vector control cells (Figures 3B,D) were injected into athymic nude mice. Both cell lines caused tumorigenesis in the athymic nude mic. Tumor growth curves showed that the growth rate of tumors that developed from the SERPIND1-overexpressing cells was significantly higher than that of the control group. At day 26 after ovarian cancer cell implantation, the average tumor weight in the SERPIND1 overexpression group was ~2.80 times that of the control group (Figure 3E). The average tumor volume in the SERPIND1 overexpression group was ~2.69 times that of the control group (Figure 3F). An autopsy revealed a number of peritoneal metastatic nodules in the SERPIND1 overexpression group (Figure 3G), whereas few metastatic nodules were detected in the control group (Figure 3H).

### Effects of SERPIND1 on the Epithelial–Mesenchymal Transition of Ovarian Cancer Cells via the PI3K/AKT Pathway

**Figure 4:**
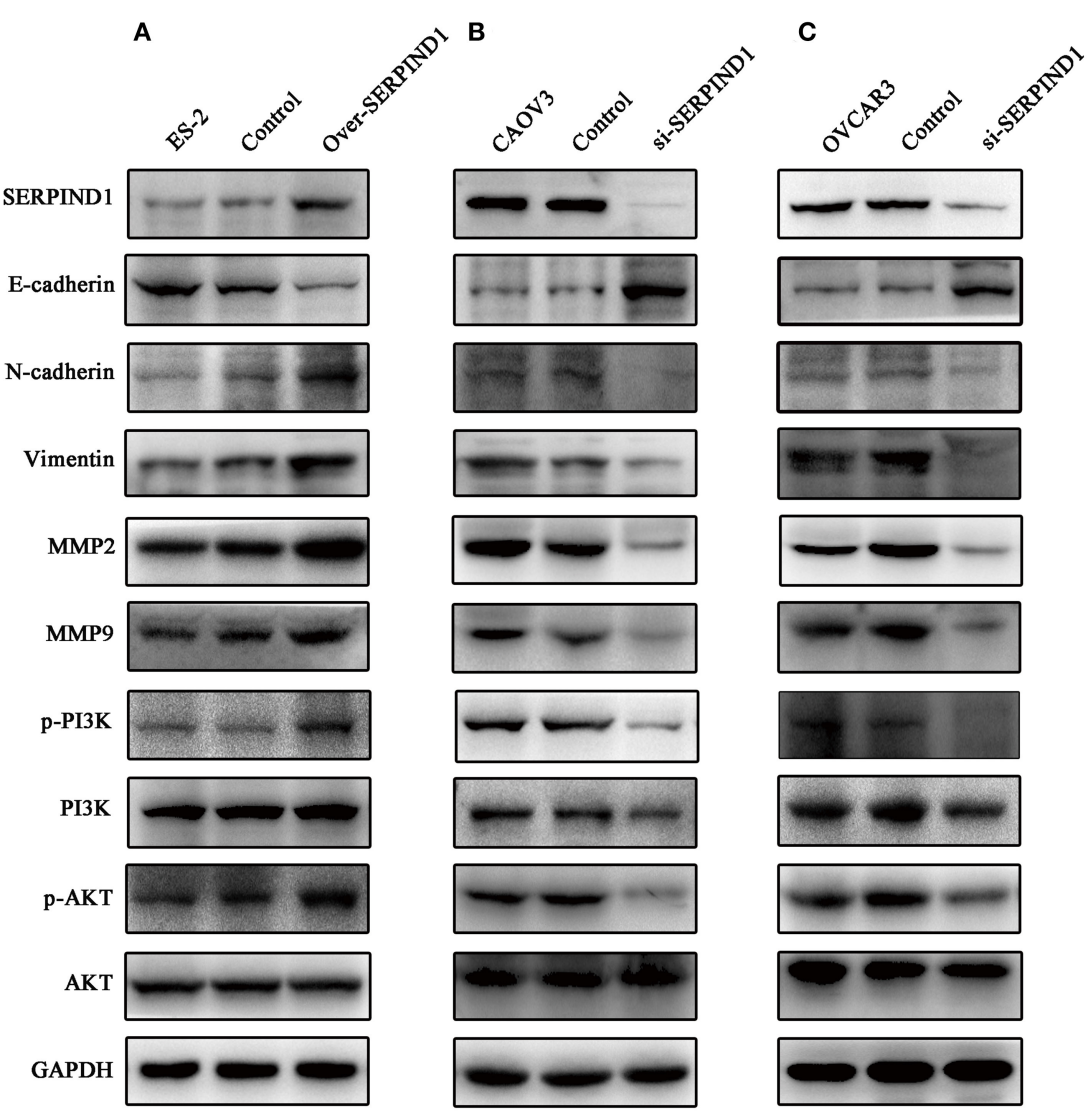
SERPIND1 regulated the EMT of ovarian cancer cells via the PI3K/AKT pathway. **(A)** SERPIND1 overexpression in ES-2 cells resulted in a significant reduction in E-cadherin expression and increased expressions of N-cadherin, Vimentin, MMP2, MMP9, p-PI3K, and p-AKT. **(B)** Inhibition of SERPIND1 expression in CAOV3 cells resulted in increased E-cadherin expression and reduced expressions of N-cadherin, Vimentin, MMP2, MMP9, p-PI3K, and p-AKT. **(C)** Inhibition of SERPIND1 expression in OVCAR3 cells resulted in increased E-cadherin expression and reduced expressions of N-cadherin, Vimentin, MMP2, MMP9, p-PI3K, and p-AKT. SERPIND1, serpin family D member 1; PI3K, phosphoinositide 3-kinase; AKT, protein kinase B.

To further analyze the role of SERPIND1 in ovarian cancer cells, we examined the major proteins of epithelial–mesenchymal transition (EMT), including MMP2, MMP9, Vimentin, N-cadherin, and E-cadherin. We noted that SERPIND1overexpression resulted in a significant reduction in E-cadherin expression and increased expressions of MMP2, MMP9, Vimentin, and N-cadherin (Figure 4A). Meanwhile, inhibition of SERPIND1 expression resulted in an increased E-cadherin expression and reduced expressions of MMP2, MMP9, Vimentin, and N-cadherin (Figures 4B,C). These results indicate that SERPIND1 promoted the migration and invasion of ovarian cancer cells through mediating EMT. The PI3K/AKT pathway is important in regulating EMT. Therefore, we examined whether SERPIND1 promoted the EMT of cells through the PI3K/AKT pathway. The results showed that SERPIND1 overexpression promoted the expressions of phospho-PI3K p85(Tyr458) and phosphor-AKT (Ser473) (i.e., increased the ratios of p-PI3K/PI3K and p-AKT/AKT) (Figure 4A), whereas inhibition of SERPIND1 reduced PI3K/AKT phosphorylation (i.e., decreased the ratios of p-PI3K/PI3K and p-AKT/AKT) (Figures 4B,C). Based on these results, we speculate that SERPIND1 regulated the EMT of ovarian cancer cells via the PI3K/AKT pathway.

### SERPIND1 Affected the Migration, Invasion, Proliferation, Apoptosis, and Other Malignant Biological Behaviors of Ovarian Cancer Cells via the PI3K/AKT Pathway

**Figure 5:**
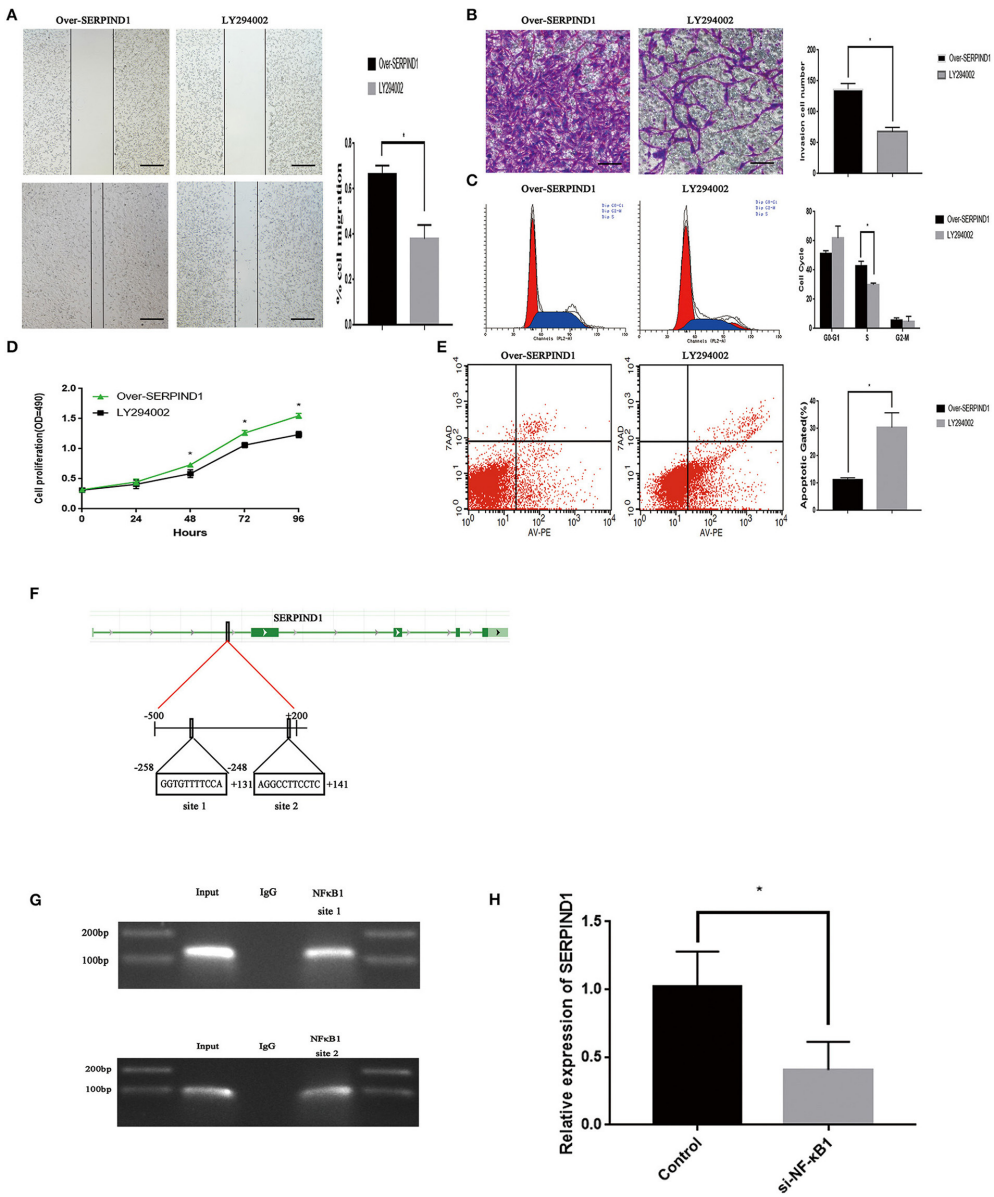
The transcription factor NF-κB1 could regulate SERPIND1 expression, which in turn could activate the PI3K/AKT signaling pathway. **(A)** The PI3K/AKT pathway inhibitor LY294002 inhibited the promoting effect of SERPIND1 on the migration capacity of ovarian cancer cells. **(B)** LY294002 inhibited the promoting effect of SERPIND1 on the invasion capacity of ovarian cancer cells. **(C)** After the addition of LY294002, the SERPIND1-overexpressing ES-2 cells exhibited a reduced proportion of S-phase cells. **(D)** LY294002 inhibited the promoting effect of SERPIND1 on the proliferation capacity of ovarian cancer cells. **(E)** After the addition of LY294002, the SERPIND1-overexpressing ES-2 cells exhibited a significantly increased apoptotic rate. **(F)** A schematic diagram showing the proposed mechanism of NF-κB1 binding to the promoter region of SERPIND1. **(G)** NF-κB1 could bind to the promoter region of SERPIND1 at the −258 to −248 (GGTGTTTTCCA) and +131 to +141 (AGGCCTTCCTC) loci. **(H)** After inhibiting NF-κB1 expression, SERPIND1 expression was also reduced. Data are presented as mean ± standard deviation from three independent experiments. *P < 0.05. Scale bar: 100 μm. NF-κB1, nuclear factor kappa B1; SERPIND1, serpin family D member 1.

The over-SERPIND1 ES-2 cells placed in McCoy's 5A medium containing 10% fetal bovine serum. Then cells were pretreated with or without LY294002 (10 μM) for 48 h. The changes in migration, invasion, proliferation, apoptosis, and cell cycle of ovarian cancer cells were monitored. The results showed that after the addition of LY294002, the SERPIND1-overexpressing ES-2 cells exhibited significantly reduced migration (Figure 5A), invasion capacities (Figure 5B), a reduced proportion of S-phase cells (Figure 5C), and reduced proliferation capacities (Figure 5D), and a significantly increased apoptotic rate (Figure 5E). Based on these results, we speculated that SERPIND1 regulated the cell migration, invasion, proliferation, apoptosis, and cell cycle of ovarian cancer cells via the PI3K/AKT pathway.

### NF-κB1 Could Bind to the Promoter Region of SERPIND1 to Regulate SERPIND1 Expression

We have also investigated the upstream regulatory mechanism of SERPIND1. We predicted that NF-κB1, FOXA1, ELK1, MAFK, and NR4A2 may be the transcription factors of SERPIND1 and regions where these transcription factors may bind to the SERPIND promoter from the JASPAR database (see Supplementary Materials). We designed the relevant primers using Primer Premier software. Then we verified by PCR whether the designed primers are available. Finally, the chromatin immunoprecipitation experiments revealed that NF- κB1 could bind to the promoter region of SERPIND1. And NF-κB1 could bind to the promoter region of SERPIND1 at the −258 to −248 (GGTGTTTTCCA) and +131 to +141 (AGGCCTTCCTC) from the transcription start loci (Figures 5F,G). Additionally, SERPIND1 expression was also reduced following the inhibition of NF-κB1 expression (Figure 5H). This demonstrates that NF-κB1 could exert a regulatory effect on SERPIND1 expression.

## Discussion

This study illustrated the effects of SERPIND1 on the malignant biological behavior of ovarian cancer cells, and showed that NF-κB1 regulated SERPIND1, which subsequently mediated cell migration, invasion, proliferation, apoptosis, cell cycle regulation, and EMT via the PI3K/AKT signaling pathway. In addition, our data showed that SERPIND1 overexpression was associated with shorter disease-free survival rates and shorter overall survival times in ovarian cancer patients. To our knowledge, this is the first study to investigate the expression and upstream regulatory mechanism of SERPIND1 in epithelial ovarian cancers.

Previous studies on SERPIND1 have focused on its ability to control vascular homeostasis and inhibit thrombin action (19, 28, 29). Another research has shown that PI3K p110β has an important role in sustaining integrin α_IIb_β_3_ activation and stable platelet aggregation by regulating both integrin α_IIb_β_3_-dependent calcium flux and G_i_-activation of Rap1b. And PI3K p110β inhibitors reduce platelet adhesion and aggregation under high shear and prevent arterial thrombotic occlusion *in vivo* (30). These findings provide a mechanistic explanation that SERPIND1 could impact in the PIK3 regulation via the thrombin system in oncogenesis. Our findings revealed that SERPIND1 was highly expressed in epithelial ovarian cancers, and the expression increased with increase in the degree of malignancy. These results suggest that SERPIND1 might be closely associated with the development and progression of epithelial ovarian cancers.

In this study, 113 patients with epithelial ovarian cancers were followed up. With sufficient data on these patients and a longer follow-up period, we could sufficiently avoid statistical bias. Our study discovered that the high expression of SERPIND1 was associated with poor prognosis through the long-term, large-sample-size follow-ups. The results showed that FIGO stage and SERPIND1 expression were risk factors of prognosis of epithelial ovarian cancers.

SERPIND1 contributes to the malignant behavior of cancer cells through involvement in diverse cellular processes. We examined SERPIND1 expression in four human ovarian cancer cell lines (CAOV3, OVCAR3, SKOV3, and ES-2) and found that SERPIND1 was expressed in all human ovarian cancer cell lines, with lower expression in ES-2 and higher expression in CAOV3 and OVCAR3. In this study, the ovarian cancer cell line ES-2 that had low SERPIND1 expression was selected as the experimental cell line for the overexpression of the *SERPIND1* gene. Compared with the control group, the SERPIND1-overexpressing ES-2 cells showed significantly increased proliferation, migration, and invasion capacities and an increased proportion of S-phase cells. Conversely, the apoptotic rate was significantly decreased.

These processes were reversed after the addition of the PI3K/AKT pathway inhibitor LY294002. The above observations indicate that PI3K is a downstream effector of SERPIND1. The ovarian cancer cell lines CAOV3 and OVCAR3 that had higher SERPIND1 expression was selected as the experimental cell lines for the suppression expression of the *SERPIND1* gene. The expression of the *SERPIND1* gene was inhibited in the ovarian cancer cell lines CAOV3 and OVCAR3, compared with the controls, and these cells showed a significant reduction in proliferation, migration, and invasion capacities, a reduced proportion of S-phase cells, and a significant increase in apoptotic rate. Based on these results, we speculated that SERPIND1 regulated the cell migration, invasion, proliferation, apoptosis, and cell cycle of ovarian cancer cells via the PI3K/AKT pathway. The most important biological characteristics of ovarian cancer cell are proliferation and metastasis. And metastasis is the leading cause of ovarian cancer deaths (31). With the experiments *in vivo*, we found that SERPIND1 could promote the ability of ovarian cancer cells for proliferation and migration.

EMT is a process by which epithelial cells lose their cell polarity and intercellular adhesion and transition into mesenchymal cells with the associated morphological characteristics, under the effects of certain physiological, pathological, and environmental factors, which enables the cells to acquire enhanced invasion capacity and motility (32). EMT plays a key role in the migration capability and invasiveness of cancer (32–34). The primary features of the EMT process include the reduction of E-cadherin expression, the increase of N-cadherin, Vimentin, and fibronectin expressions (32, 35, 36), conversion of cytokeratin and cytoskeletal proteins into Vimentin-based cytoskeleton, and display of morphological characteristics of mesenchymal cells (32–39). These changes lead to weak intercellular interactions that favor the migration capability of cells (32, 40). Additionally, N-cadherin regulates the activity of specific receptor tyrosine kinase pathways, thereby promoting cell removal (32). Cells undergoing EMT loosely interact with the basement membrane (40, 41), which could lead to cells regulating the expressions of specific integrins and matrix metalloproteinases (42–44). Moreover, cells acquire the ability to interact with different extracellular matrices over time (41–46). Another study has found that the PI3K/AKT signaling pathway can influence EMT in a number of ways to affect cancer motility and aggressiveness (47).

In this study, we have found for the first time that SERPIND1 overexpression in ovarian cancer cells resulted in increased expression of phospho-PI3K p85 and phosphor-AKT (Ser473), with concurrent significant reduction in E-cadherin expression and increased expressions of N-cadherin, Vimentin, MMP2, and MMP9. In contrast, the inhibition of SERPIND1 expression in ovarian cancer cells reduced the phosphorylation of PI3K/AKT, increased E-cadherin expression, and reduced the expressions of N-cadherin, Vimentin, MMP2, and MMP9. Based on these results, we speculate that SERPIND1 promoted the proliferation, migration, invasion, G1-to-S phase transition, and EMT of ovarian cancer cells as well as inhibited their apoptosis via the PI3K/AKT pathway. Thus, SERPIND1 may serve as a potential target for ovarian cancer therapy.

The transcription factor NF-κB, which was first extracted from B cells and named by Sen et al. (48), is a class of nuclear proteins with diverse regulatory effects. NF-κB is widely present in eukaryotic cells for the control of DNA transcription. In resting cells, NF-κB binds to an inhibitor and remains in the cytoplasm. When the cells are subjected to external stimuli or stress, they will activate the corresponding signaling pathway, which in turn activates the IκB kinase that can phosphorylate and degrade the inhibitor IκBa. The released NF- κB1 can then enter the nucleus and exert its transcription factor function. NF-κB1 is a protein encoded by the NF-κB1 gene and processed by proteasomes to form NF-κBp50. NF-κBp50 is the DNA-binding subunit of the NF-κB protein complex. NF-κBp50 binds to a fixed nucleotide sequence in the gene promoter region to initiate gene transcription and induce target gene expression, through which it participates in a series of important biological processes including immunity, inflammation, oxidative stress, cell proliferation, apoptosis, tumorigenesis, and metastasis (49). We have also investigated the upstream regulatory mechanism of SERPIND1. We predicted that NF-κB1, FOXA1, ELK1, MAFK, and NR4A2 may be the transcription factors of SERPIND1 and regions where these transcription factors may bind to the SERPIND promoter from the JASPAR database. Chromatin immunoprecipitation experiments showed that NF-κB1 could bind to the Promoter region of SERPIND1, and the binding site was located at the −258 to −248 (GGTGTTTTCCA) and +131 to +141 (AGGCCTTCCTC) loci of the SERPIND1 promoter region. Moreover, the inhibition of NF-κB1 expression reduced the SERPIND1 expression, indicating that NF-κB1 exerted a regulatory effect on SERPIND1 expression.

However, this study has many limitations. Due to limited conditions, some patients had not undergone lymph node dissection, so the conclusions drawn from the results could have limitations. Furthermore, this study has not conducted a detailed experiment on how PI3K/AKT affects EMT. Thus, we are planning to complete the experiment in our next study.

**Figure 6:**
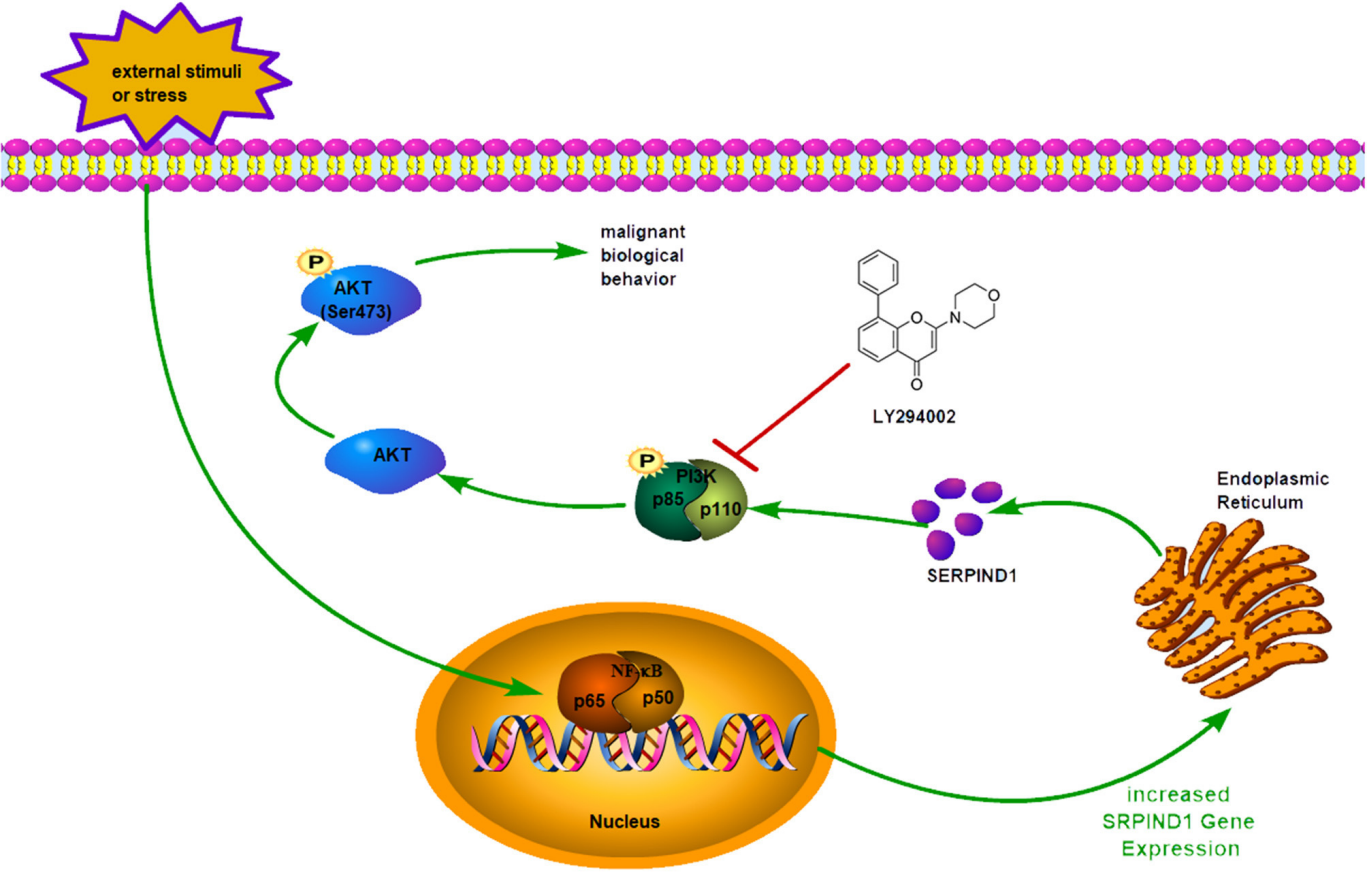
Schematic model showing: When the cells are subjected to external stimuli, NF-κB1 could enter the nucleus and exert its transcription factor function. The transcription factor NF-κB1 could bind to the promoter region of SERPIND1 and regulate SERPIND1 expression, which in turn could affect the PI3K/AKT pathway and promote the malignant biological behavior of ovarian cancer cells.

In conclusion, we found that SERPIND1 was overexpressed in epithelial ovarian cancer, and such overexpression predicted a poor prognosis of the disease. In addition, the transcription factor NF-κB1 could bind to the promoter region of SERPIND1 and regulate SERPIND1 expression, which in turn could affect the PI3K/AKT pathway and promote the malignant biological behavior of ovarian cancer cells (Figure 6). Our results ultimately showed that SERPIND1 could be an effective early diagnostic and prognostic marker for epithelial ovarian cancers and potentially serve as a novel drug target.

## Data Availability Statement

All datasets generated for this study are included in the manuscript/ Supplementary Files.

## Ethics Statement

The use of nude mice in this study was approved by the Medical Ethics Committee of the Shengjing Hospital of China Medical University (2018PS106K). Pathological analysis was approved by the Ethics Review Committee of the China Medical University (2010PS84K).

## Author Contributions

QG conceived and designed the study, performed the experiments, and drafted the manuscript. LZ, CW, and SW helped to analyze the data. XN evaluated and scored the immunohistochemistry staining. JL, QL, YH, and XL were involved in coordination of the work. BL conceived and supervised the study. All authors have reviewed and approved the final manuscript.

### Conflict of Interest

The authors declare that the research was conducted in the absence of any commercial or financial relationships that could be construed as a potential conflict of interest.
