# Corrigendum: SERPIND1 affects the malignant biological behavior of epithelial ovarian cancer via the PI3K/AKT pathway: a mechanistic study

**DOI:** 10.3389/fonc.2024.1466959

**Published:** 2024-12-03

**Authors:** Qian Guo, Liancheng Zhu, Caixia Wang, Shuang Wang, Xin Nie, Juanjuan Liu, Qing Liu, Yingying Hao, Xiao Li, Bei Lin

**Affiliations:** ^1^ Department of Obstetrics and Gynecology, Shengjing Hospital of China Medical University, Shenyang, China; ^2^ Key Laboratory of Maternal-Fetal Medicine of Liaoning Province, Benxi, China; ^3^ Key Laboratory of Obstetrics and Gynecology of Higher Education of Liaoning Province, Benxi, China

**Keywords:** epithelial ovarian cancer, SERPIND1, prognosis, proliferation, cell cycle, cell migration, EMT, NF-κB1

In the published article, there was an error in **Figure 2B** as published. The image for panel (8) was incorrectly placed, resulting in a repetition of the image for panel (7). Consequently, both panels (7) and (8) show the OVCAR3 transwell image, while the OVCAR3-SERPIND1-L-Mock transwell image was not included in the correct position. The correct content should have panel (7) showing the OVCAR3 transwell image and panel (8) showing the blank control OVCAR3-SERPIND1-L-Mock transwell image. This was entirely an inadvertent error due to the similar naming of the images, leading to the incorrect image being used. The corrected **Figure 2B** and its caption appear below.

**Figure 2:**
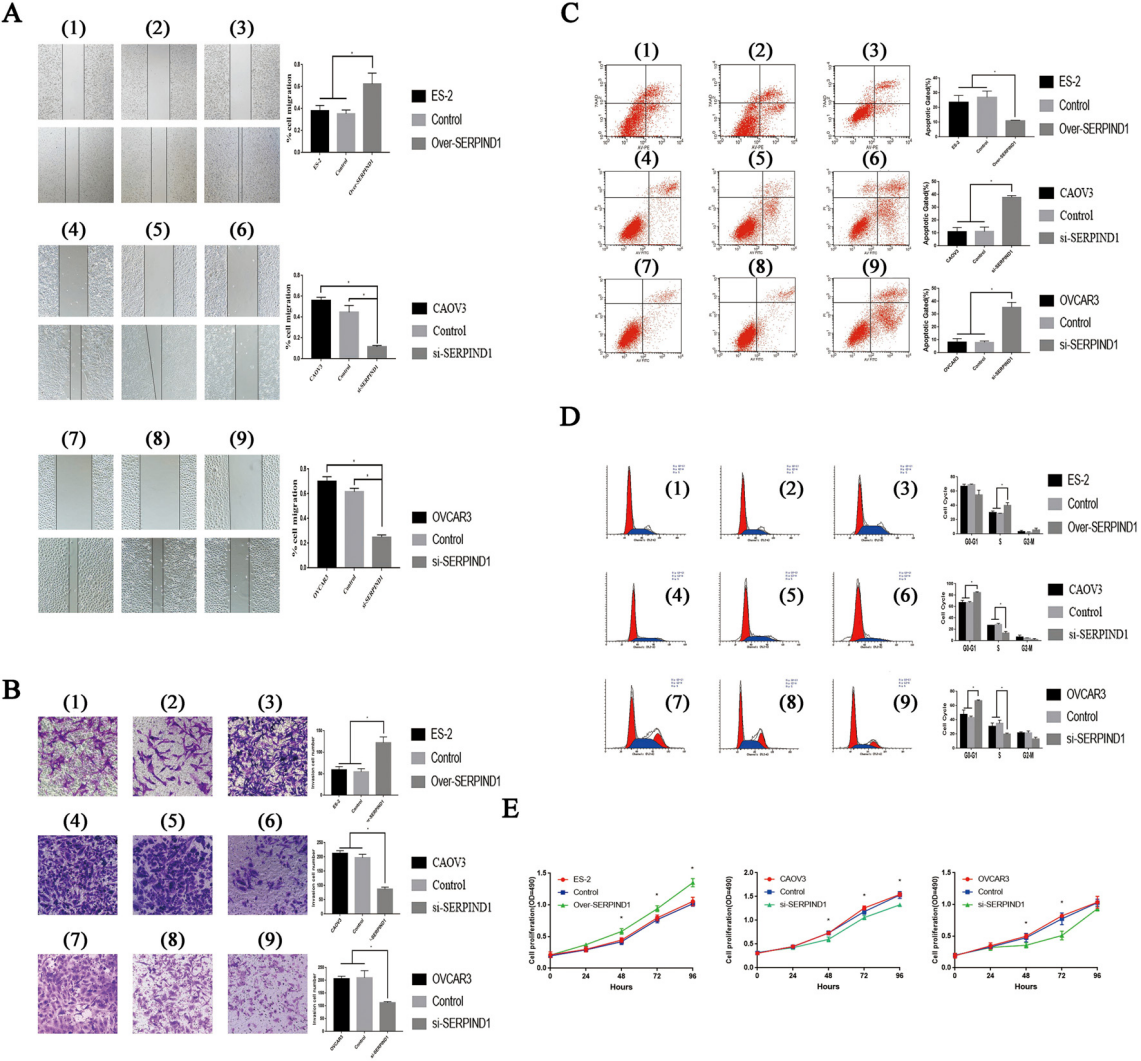
Effects of SERPIND1 on ovarian cancer cells *in vitro*. **(A)** SERPIND1 promoted the migration capacity of ovarian cancer cells. **(B)** SERPIND1 promoted the invasion capacity of ovarian cancer cells. **(C)** SERPIND1 inhibited apoptosis in ovarian cancer cell lines. **(D)** Effects of SERPIND1 on the cell cycle of ovarian cancer cell lines. **(E)** SERPIND1 promoted the proliferation of ovarian cancer cells. (1): ES-2; (2): ES-2-SERPIND1-H-Mock; (3): ES-2-SERPIND1-H; (4): CAOV3; (5): CAOV3-SERPIND1-L- Mock; (6): CAOV3-SERPIND1-L; (7): OVCAR3; (8): OVCAR3-SERPIND1-L- Mock (9): OVCAR3-SERPIND1-L. Data are presented as mean ± standard deviation from three independent experiments. *P < 0.05. Scale bar: 100 μm. SERPIND1, serpin family D member 1.

The authors apologize for this error and state that this does not change the scientific conclusions of the article in any way. The original article has been updated.

