# Supplementary material for: SERPIND1 Affects the Malignant Biological Behavior of Epithelial Ovarian Cancer via the PI3K/AKT Pathway: A Mechanistic Study
Source: Front Oncol. 2019 Oct 4;9:954. doi: 10.3389/fonc.2019.00954 (PMC6788328; doi:10.3389/fonc.2019.00954)
Supplement: Supplementary file 1 [file Data_Sheet_1.docx]

**The Methods Of Western Blot.**

Ice-cold RIPA buffer was added to ovarian cancer cells and tissues separately, followed by incubation at 4°C for 40 min. After centrifugation at 12,000 × g for 30 min at 4°C. Supernatant fractions were collected. Add 1/4 volume of 5×loading buffer to the supernatant fractions. Protein concentration was measured using Bicinchoninic acid (23228, Thermo Fisher Scientific, Waltham, MA, USA). Equal amounts of protein from each sample were separated by electrophoresis on SDS-10% polyacrylamide gel. The proteins were transferred to PVDF membrane (0.45um, Millipore, USA) and blocked with 5% BSA diluted in 1% TBS added with 0.1% Tween 20 at room temperature for 2 hours. The membrane was incubated with primary antibody in 1 x TBS, 0.1% Tween 20 overnight at 4°C.

The primary antibodies used in our study were as follows:

SERPIND1 (1:1000 dilution; Abcam, Cambridge, UK);

E-cadherin, N-cadherin, MMP2 and MMP9 (1:1000 dilution; Proteintech Group Inc., USA); Vimentin (1:1500 dilution; Proteintech Group Inc., USA);

And PI3K p85, phospho-PI3K p85(Tyr458)/p55(Tyr199), AKT (pan), and phosphor-AKT (Ser473) (1:1000 dilution; Cell Signaling Technology, Danvers, MA, USA); GAPDH (1:3000 dilution; Zhong Shan Company, China).

The PVDF membranes were washed and incubated again for 2 h at room temperature with horseradish-peroxidase-conjugated anti-rabbit or anti-mouse secondary antibody. The proteins were visualized with ECL reagent (ECL Prime Western Blotting Detection Reagent, Amersham, Pittsburgh, PA, USA). The experiments were repeated three times.
