# Supplementary material for: SERPIND1 Affects the Malignant Biological Behavior of Epithelial Ovarian Cancer via the PI3K/AKT Pathway: A Mechanistic Study
Source: Front Oncol. 2019 Oct 4;9:954. doi: 10.3389/fonc.2019.00954 (PMC6788328; doi:10.3389/fonc.2019.00954)
Supplement: Supplementary file 2 [file Data_Sheet_2.docx]

Possible transcription factors of SERPIND1 and regions where these transcription factors may bind to the SERPIND promoter from the JASPAR database

| Model ID | Model name | Score | Relative score | Start | End | Strand | Predicted site sequence |
| --- | --- | --- | --- | --- | --- | --- | --- |
| MA0148.3 | FOXA1 | 9.681 | 0.899432641719189 | 1477 | 1491 | -1 | CTTATGTGTGCACTG |
| MA0105.3 | NFKB1 | 8.811 | 0.877513245137574 | 1743 | 1753 | 1 | GGTGTTTTCCA |
| MA0028.1 | ELK1 | 8.789 | 0.904316624661268 | 173 | 182 | 1 | GTGCCTGAAG |
| MA0148.3 | FOXA1 | 7.614 | 0.876330578710687 | 915 | 929 | -1 | TCTGTATTTGTACAT |
| MA0148.3 | FOXA1 | 7.129 | 0.870909920481793 | 1310 | 1324 | 1 | CCGTTGTATACTCAG |
| MA0105.3 | NFKB1 | 7.002 | 0.851122590240519 | 1742 | 1752 | 1 | GGGTGTTTTCC |
| MA0105.3 | NFKB1 | 6.219 | 0.839699769464182 | 725 | 735 | -1 | CGGGGTGTCCC |
| MA0496.1 | MAFK | 6.212 | 0.840075228563519 | 1184 | 1198 | -1 | TCCTGTCATCACCTA |
| MA0160.1 | NR4A2 | 6.212 | 0.809453437591938 | 1288 | 1295 | 1 | CAGATCAG |
| MA0160.1 | NR4A2 | 6.212 | 0.809453437591938 | 1472 | 1479 | 1 | CAGATCAG |
